# Magnitude Estimation with Noisy Integrators Linked by an Adaptive Reference

**DOI:** 10.3389/fnint.2016.00006

**Published:** 2016-02-16

**Authors:** Kay Thurley

**Affiliations:** ^1^Department Biology II, Ludwig-Maximilians-Universität MünchenMünchen, Germany; ^2^Bernstein Center for Computational NeuroscienceMunich, Germany

**Keywords:** magnitude estimation, interval timing, drift-diffusion model, uncertainty, regression effect, range effect, optimality

## Abstract

Judgments of physical stimuli show characteristic biases; relatively small stimuli are overestimated whereas relatively large stimuli are underestimated (regression effect). Such biases likely result from a strategy that seeks to minimize errors given noisy estimates about stimuli that itself are drawn from a distribution, i.e., the statistics of the environment. While being conceptually well described, it is unclear how such a strategy could be implemented neurally. The present paper aims toward answering this question. A theoretical approach is introduced that describes magnitude estimation as two successive stages of noisy (neural) integration. Both stages are linked by a reference memory that is updated with every new stimulus. The model reproduces the behavioral characteristics of magnitude estimation and makes several experimentally testable predictions. Moreover, the model identifies the regression effect as a means of minimizing estimation errors and explains how this optimality strategy depends on the subject's discrimination abilities and on the stimulus statistics. The latter influence predicts another property of magnitude estimation, the so-called range effect. Beyond being successful in describing decision-making, the present work suggests that noisy integration may also be important in processing magnitudes.

## 1. Introduction

**Figure 1:**
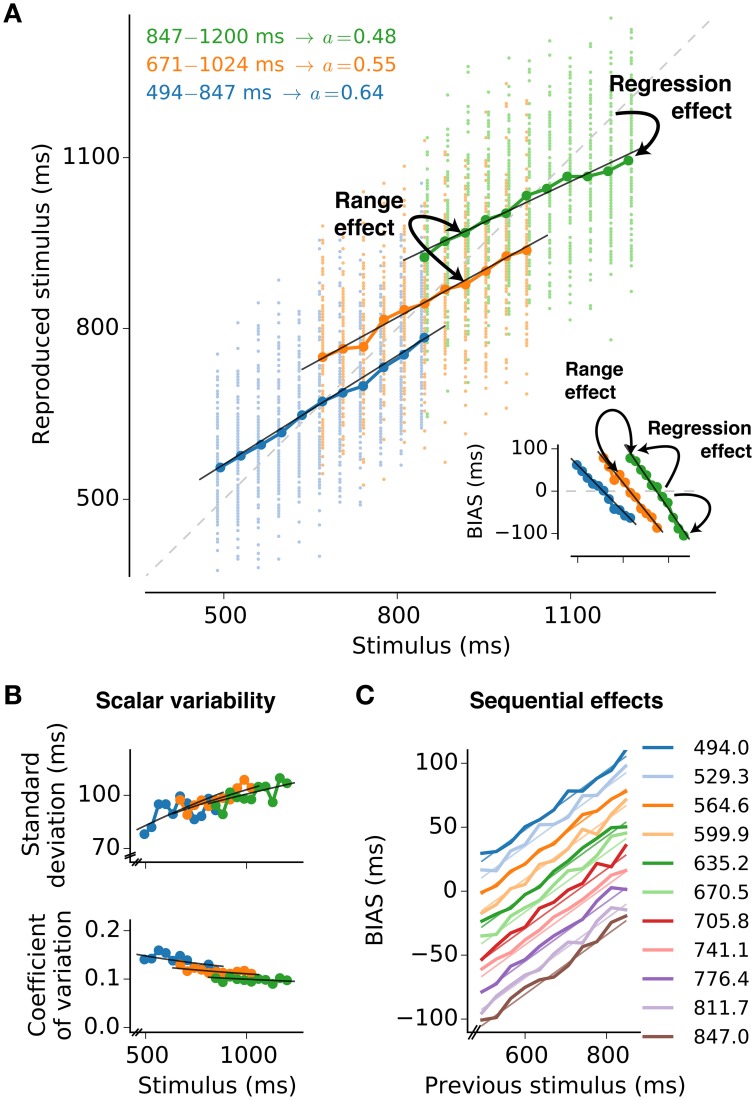
**Psychophysical characteristics of magnitude estimation**. The typical properties of magnitude estimation are illustrated as they are reproduced by the model presented in this paper. The description is based on subsecond interval timing (cf., Jazayeri and Shadlen, 2010). **(A)** Individual reproduced values for each trial and stimulus (small dots, 100 per stimulus value), and their averages (large circles connected by lines) are shown for a simulation with three stimulus ranges. The regression effect is the deviation of the averages from the line of equality (diagonal gray dashed line) toward the mean of the respective stimulus range. It becomes stronger with larger means of the stimulus range, i.e., range effect. The analytical approximation of the model is in line with the simulated data (black solid lines). The memory parameter *a* was chosen to minimize MSE_*r*_ for each range (derived in Section “3.1”). Stimulus ranges and memory weights *a* are given in the top-left corner of the plot. Other parameters are *A*_*m*_ = *A*_*r*_ = 0.25, σ_*m*_ = 1, and σ_*r*_ = 0.5. *Inset:* Average deviations (BIAS) from the line of equality for each stimulus and test range. Solid lines are again analytical predictions. **(B)** Standard deviation and coefficient of variation (standard deviation divided by the mean) corresponding to **(A)**. Black solid lines are again analytical predictions. **(C)** Sequential effects. Plotting the response bias for a certain stimulus as a function of the stimulus in the previous trial, reveals effects of stimulus order in the simulations (thick lines). The simulation results can be analytically approximated (thin lines). Results for the range 494 − 847 ms are displayed. For each stimulus value 10,000 trials were simulated.

In daily life we continuously need to process the physical conditions of our environment; we make judgements about the magnitude of sensory stimuli, represent them neurally and base decisions upon them. Judgements about magnitudes are inherently unreliable due to noise from different sources such as the statistics of the physical world, the judgement process itself, the neural representation of the stimulus and finally the computations that drive behavior. A large body of experimental work highlights that magnitude estimation is subject to characteristic psychophysical effects. These effects are strikingly similar across different sensory modalities, suggesting common processing mechanisms that are shared by different sensory systems (for a recent review see Petzschner et al., 2015). Amongst the behavioral characteristics the most astonishing yet unresolved is the *regression effect* also known as regression to the mean, central tendency, or Vierordt's law (von Vierordt, 1868; Hollingworth, 1910; Shi et al., 2013). It states that over a range of stimuli, small stimuli are overestimated whereas large stimuli are underestimated (Figure 1A). Regression becomes more pronounced for ranges that comprise larger stimulus values (*range effect*; Teghtsoonian and Teghtsoonian, 1978). As a consequence the same stimuli lead to different responses on average when embedded in different but overlapping stimulus distributions (Figure 1A) — the responses depend on the stimulus context (Jazayeri and Shadlen, 2010). Another omnipresent effect in magnitude estimation experiments is *scalar variability*, i.e., errors monotonically increase with the size of the stimulus, attributed to the famous Weber-Fechner law Figure 1B; (Weber, 1851; Fechner, 1860). Finally, magnitude estimation is influenced by the sequence in which stimuli are presented (Cross, 1973; Hellström, 2003; Dyjas et al., 2012). According to such *sequential effects* the estimate of the stimulus in a particular trial is affected by the previous trial. This results in under- or overestimation of the current stimulus depending on the previous stimulus (Figure 1C).

The above behavioral characteristics likely result from an optimal strategy when noisy estimates are made about stimuli that itself depend on the statistics of the environment. Recently such optimality strategies were successfully explained in Bayesian frameworks (Jazayeri and Shadlen, 2010; Petzschner and Glasauer, 2011; Cicchini et al., 2012). Bayesian models incorporate a-priori knowledge about the stimuli into the estimation process, which seems to be crucial in explaining the aforementioned behavioral phenomena. However, the cited Bayesian approaches represent conceptual descriptions; inference about brain implementation is challenging.

The present paper introduces a theoretical approach that formulates magnitude estimation with noisy integrators (drift-diffusion processes). The model comprises two successive stages, measurement and reproduction. During measurement the current stimulus is estimated via noisy integration. The estimate is then combined with information from previous trials and used as threshold in the reproduction stage. The first passage of the threshold during reproduction determines the magnitude of the reproduced stimulus. Since the threshold depends on both the current and previous trials, it acts as an internal reference memory that is updated with every new stimulus. As we will see below, the model reproduces the behavioral characteristics of magnitude estimation (Figure 1 anticipates these results) and interprets them as a consequence of an optimization strategy to minimize reproduction errors given noisy estimates and stimulus statistics.

## 2. Materials and methods

The analytical methods employed in this paper rely on standard mathematical and statistical techniques. Simulation and numerical analysis was performed with Python 2.7 using the packages: Numpy 1.9, Scipy 0.15, Statsmodels 0.6 (Seabold and Perktold, 2010), and Matplotlib 1.4 (Hunter, 2007). The model's stochastic differential equations, Equations (1, 4), were simulated via the approximation

$$\begin{array}{l} x_{i+1}=x_{i}+A\Delta t+\sigma \sqrt{\Delta t}\cdot N\left(0,1\right)\;. \end{array}$$

A time step Δ*t* = 5 ms was used, to appropriately sample the Gaussian process N(0, 1), and capture noise sources on fast time scales like sensory noise and irregular spiking dynamics, at reasonable computing times.

### 2.1. Definition of the model

**Figure 2:**
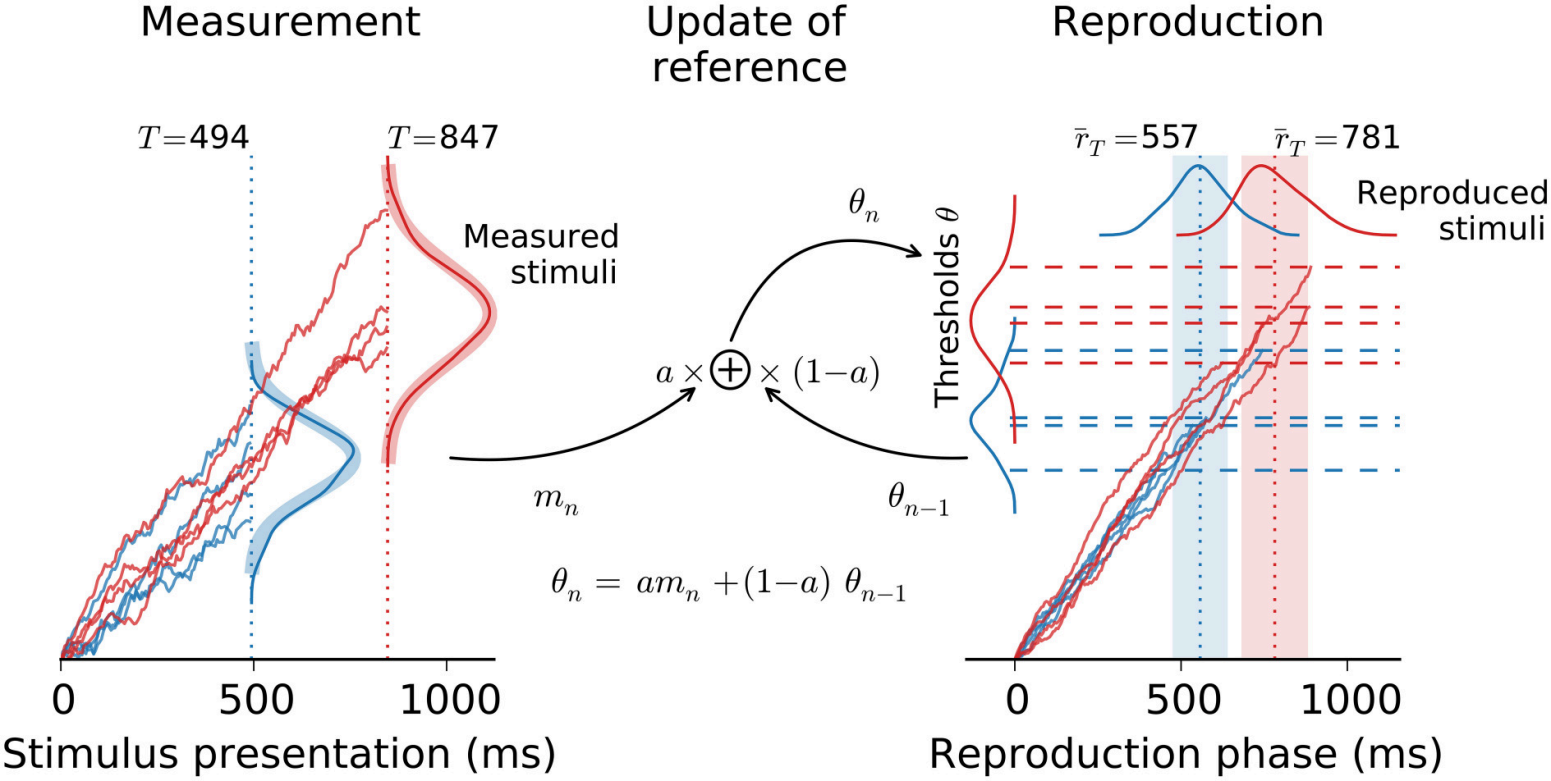
**Architecture of the model**. The model comprises the measurement of the stimulus followed by its reproduction. Both stages are connected via the threshold θ for the reproduction stage (dashed lines), which combines the measurement of the current stimulus *m*_*n*_ with the threshold θ_*n*−1_ from the previous trial, i.e., the reference. Example traces are displayed for intervals of 494 ms (blue) and 847 ms (red). Kernel density estimates are provided for the distributions of the model's stochastic variables (derived from 100 simulation runs for each stimulus in the range 49 4− 847 ms from Figure 1). Thick shaded lines in the measurement stage are theoretical distributions. Dotted vertical lines and shaded areas in the reproduction stage give predicted mean ± std.

Estimating the magnitude of a stimulus comprises two stages: First the stimulus is measured and afterwards the measurement is reported, e.g., reproduced by matching the strength of the stimulus. In the present paper, both measurement and reproduction are modeled as drift-diffusion processes (e.g., Bogacz et al., 2006). During measurement drift-diffusion is left running as long as the stimulus is presented. Whereas, in the reproduction stage the drift-diffusion process is not stopped from outside but lasts until it hits a threshold from below. This threshold depends on the stimulus as estimated in the measurement stage and also includes the history of thresholds from previous trials, serving as an internal reference. Figure 2 gives an overview of the model. For simplicity, the description below focuses on the estimation of temporal intervals (*interval timing*; Merchant et al., 2013). Numbers refer to interval timing in the subsecond range after Jazayeri and Shadlen (2010). However, application to the estimation of, e.g., sound intensity or spatial distances, is straightforward by reinterpreting the variables accordingly.

#### 2.1.1. Measurement

The measurement stage comprises a drift process with rate *A*_*m*_ that is corrupted by noise (diffusion) realized as a Wiener process *W* with an amplitude σ_*m*_. The dynamics are described by a stochastic differential equation

(1) $$\begin{array}{l} dm=A_{m}dt+\sigma_{m}dW,m\left(0\right)=0\;. \end{array}$$

The process is assumed to finish with the end of the stimulus and its final state yields the measurement. We can calculate the latter by integrating the above formula between stimulus start at *t* = 0 and end at *t* = *T* (Broderick et al., 2009) and obtain

(2) $$\begin{array}{l} m_{T}:=m\left(T\right)=A_{m}T+\sigma_{m}\sqrt{T}\cdot N\left(0,1\right)\;. \end{array}$$

For convenience let us write *m*_*T*_ when we are considering a trial in which the interval *T* was presented, i.e., *m*(*T*). The final value *m*_*T*_ of the measurement process is Gauss-distributed $m_{T}\sim N\left(A_{m}T,\sigma_{m}^{2}T\right)$ with mean ${\bar{m}}_{T}=E\left(m|T\right)=A_{m}T$ and variance $\mathrm{Var}\left(m_{T}\right)=\mathrm{Var}\left(m|T\right)=\sigma_{m}^{2}T$. This value is incorporated into the threshold of the reproduction phase as detailed below.

For later use, let us also derive the overall variance of the measurement Var (*m*) here. To calculate Var (*m*), we apply the law of total variance and get

(3) $$\begin{array}{l} \mathrm{Var}\left(m\right)=E\left(\mathrm{Var}\left(m|T\right)\right)+\mathrm{Var}\left(E\left(m|T\right)\right)\\ =E\left(\sigma_{m}^{2}T\right)+\mathrm{Var}\left(A_{m}T\right)\\ =\sigma_{m}^{2}E\left(T\right)+A_{m}^{2}\mathrm{Var}\left(T\right)\;. \end{array}$$

#### 2.1.2. Reproduction

Similarly to the measurement stage, reproduction is modeled as drift-diffusion with corresponding drift *A*_*r*_ and noise amplitude σ_*r*_. However, here, the process is not stopped after a certain time but limited by an upper bound, i.e., a threshold θ (Broderick et al., 2009),

(4) $$\begin{array}{l} dr=A_{r}dt+\sigma_{r}dW,r\left(0\right)=0\text{ and }r\left(t\right)<\theta \;. \end{array}$$

The time of threshold crossing from below, i.e., the first-passage time of the drift-diffusion process, represents the response or the reproduced stimulus interval, respectively. Since we have a drift-diffusion process with a single threshold θ > 0, the distribution of its first-passage times has an inverse Gaussian density $IG\left(\mu ,\lambda \right):f\left(x;\mu ,\lambda \right)={\left[\frac{\lambda }{2\pi x^{3}}\right]}^{1∕2}\exp\left(-\frac{\lambda {\left(x-\mu \right)}^{2}}{2\mu^{2}x}\right)$ and is characterized by *X* ~ *IG*(μ, λ) : E (*X*) = μ, Var (*X*) = μ^3^∕λ (Tuckwell, 1988). In the present case, we have (cf. Simen et al., 2011):

(5) $$\begin{array}{l} r_{T}\sim IG\left(\frac{\theta_{T}}{A_{r}},\frac{\theta_{T}^{2}}{\sigma_{r}^{2}}\right):{\bar{r}}_{T}\;:=\;E\left(r|T\right)=\frac{\theta_{T}}{A_{r}},\\ \mathrm{Var}\left(r_{T}\right)\;:=\;\mathrm{Var}\left(r|T\right)=\frac{\theta_{T}\sigma_{r}^{2}}{A_{r}^{3}}\;. \end{array}$$

The reproduced stimulus interval that corresponds to the presentation of a stimulus *T* is denoted by *r*_*T*_ and θ_*T*_ is the threshold in this trial.

#### 2.1.3. Threshold for reproduction

As already mentioned above, in a trial *n* the threshold θ_*n*_ in the reproduction stage depends on the stimulus' measurement *m*_*n*_ and the threshold θ_*n*−1_ of the previous trial

(6) $$\begin{array}{l} \theta_{n}=am_{n}+\left(1-a\right)\theta_{n-1},\theta_{0}=m_{0}\;. \end{array}$$

The weight *a* is limited to the interval 0 < *a* ≤ 1. A value of *a* = 0 has to be excluded since for *a* = 0 only the first stimulus would be taken into account and has an everlasting memory. The formulation in Equation (6) effectively introduces a weighted average preventing unbounded growth. A reference memory is formed and updated on a single trial basis. As we will see later, the memory weight *a* has an immediate impact on the relation between stimulus and response. The recursive definition in Equation (6) can also be given as an iterative formula

(7) $$\begin{array}{l} \theta_{n}=am_{n}+a\overset{n-1}{\underset{i=1}{\sum }}{\left(1-a\right)}^{n-i}m_{i}+{\left(1-a\right)}^{n}m_{0}\;. \end{array}$$

#### 2.1.4. Further conditions for the model

It is assumed that drifts *A*_*m*_ and *A*_*r*_ are positive numbers. In addition, the drift-diffusion processes are supposed to act in drift-dominated regimes with $A_{m∕r}T>\sigma_{m∕r}\sqrt{T}\cdot N\left(0,1\right)$. Otherwise, the measurement stage may yield negative values, resulting in negative thresholds θ, which can not be hit from below. For the sake of simplicity and without loss of generality, the model is not formulated with a lower bound that only allows for positive values. An account of the influence of a lower bound on the first passage time distribution of a drift-diffusion process can be found in Simen et al. (2011).

### 2.2. Analytical approximations

Reproduced stimuli in the model are random variables drawn from the distribution of first passage times in the reproduction stage (Equation 5). Determining the distribution of these first passage times *p*(*r*_*T*_) is complicated since the threshold θ_*T*_ itself is a random variable. Obtaining *p*(*r*_*T*_) would thus require calculating *p*(*r*_*T*_) = ∫ *dθ*_*T*_*p*(*r*_*T*_ ∣ θ_*T*_)*p*(θ_*T*_) for which a general solution can not be provided. For a Gaussian threshold distribution the calculations are exemplified by Simen et al. (2011), resulting in smeared-out inverse Gaussian distributions. Qualitatively this results also holds true for other “reasonable” threshold distributions (cf. Figure 2). To provide generic analytical solutions for the present model, the section below focuses on expected values and variances.

#### 2.2.1. Expected value of the threshold

With randomized stimulus presentations and sufficiently large numbers of preceding trials, we obtain the expected value of the threshold in the current trial from Equation (7)

(8) $$\begin{array}{l} E\left(\theta_{n}\right)=am_{n}+a\langle m\rangle \overset{n-1}{\underset{i=1}{\sum }}{\left(1-a\right)}^{n-i}+{\left(1-a\right)}^{n}m_{0}\;, \end{array}$$

with 〈·〉 denoting the trial average. The sum in Equation (8) is a geometric series and can be rewritten to

$$\begin{array}{l} E\left(\theta_{n}\right)=am_{n}+a\langle m\rangle \left[\frac{1-{\left(1-a\right)}^{n}}{1-\left(1-a\right)}-1\right]+{\left(1-a\right)}^{n}m_{0}\\ =am_{n}+\langle m\rangle \left[1-a-{\left(1-a\right)}^{n}\right]+{\left(1-a\right)}^{n}m_{0}\;. \end{array}$$

We further simplify by taking the limit *n* → ∞ and get ${\bar{\vartheta }}_{n}\;:=\;\lim_{n\to \infty }E\left(\theta_{n}\right)=am_{n}+\left(1-a\right)\langle m\rangle$. From the last expression we derive the expected value of the threshold in a trial in which the interval *T* was presented, i.e., ${\bar{\vartheta }}_{T}=E\left(\vartheta |{\bar{m}}_{T}\right)=E\left(\vartheta |T\right)$. Using 〈*m*〉 = *A*_*m*_ 〈 *T*〉 and $m_{n}={\bar{m}}_{T}=A_{m}T$, we end up with

(9) $$\begin{array}{l} {\bar{\vartheta }}_{T}=aA_{m}T+\left(1-a\right)A_{m}\langle T\rangle \;. \end{array}$$

Note, that the average threshold ${\bar{\vartheta }}_{T}$ depends on both the current stimulus *T* and the trial average 〈*T*〉. The latter is equal to the mean of the stimulus distribution 〈*T*〉 = E (*T*). The description below therefore uses E (*T*) instead of 〈*T*〉.

#### 2.2.2. Variance of the threshold

The above calculations only gave the mean threshold for a particular trial. In a next step let us derive from Equation (7) the corresponding variance. Calculating the variance of Equation (7) we obtain a slightly more elaborate geometric series

$$\begin{array}{l} \mathrm{Var}\left(\theta_{n}\right)=a^{2}\;\mathrm{Var}\left(m_{n}\right)+a^{2}\;\mathrm{Var}\left(m\right)\overset{n-1}{\underset{j=1}{\sum }}{\left(1-a\right)}^{2j}\\ \mathrm{Var}\left(\theta_{n}\right)=a^{2}\;\mathrm{Var}\left(m_{n}\right)+a^{2}\;\mathrm{Var}\left(m\right)\frac{{\left(1-a\right)}^{2}-{\left(1-a\right)}^{2n}}{1-{\left(1-a\right)}^{2}}\;. \end{array}$$

Taking the limit *n* → ∞, yields

$$\begin{array}{l} \mathrm{Var}\left(\vartheta_{n}\right)\;:=\;\underset{n\to \infty }{\lim}\mathrm{Var}\left(\theta_{n}\right)=a^{2}\;\mathrm{Var}\left(m_{n}\right)+\frac{a{\left(1-a\right)}^{2}}{2-a}\mathrm{Var}\left(m\right)\;. \end{array}$$

From the last expression we determine the variance of the threshold in a trial with stimulus interval *T*, i.e., Var (ϑ_*T*_). The variance Var (*m*_*n*_) is given by the variance of the current measurement $\mathrm{Var}\left(m_{T}\right)=\sigma_{m}^{2}T$, see Equation (2), and Var (*m*) is given by Equation (3), i.e., $\mathrm{Var}\left(m\right)=A_{m}^{2}\mathrm{Var}\left(T\right)+\sigma_{m}^{2}E\left(T\right)$. Insertion into the above formula yields

(10) $$\begin{array}{l} \mathrm{Var}\left(\vartheta_{T}\right)=a^{2}\sigma_{m}^{2}T+\left[A_{m}^{2}\mathrm{Var}\left(T\right)+\sigma_{m}^{2}E\left(T\right)\right]\frac{a{\left(1-a\right)}^{2}}{2-a}\;. \end{array}$$

Thus, similarly to the average threshold (9) its variance also depends on both the current stimulus *T* and the mean of the stimulus distribution E (*T*). A third influence comes from the variance of the stimuli Var (*T*).

#### 2.2.3. Expected value and variance for the reproduction

We can use the solutions for expected value and variance of the threshold ϑ_*T*_ from Equations (9, 10) to extend the formulas for the expected value and variance during reproduction in Equation (5). To determine the average reproduced value ${\bar{r}}_{T}\;:=\;E\left(r|T\right)$ for a stimulus *T*, we apply the law of total expectation and obtain

(11) $$\begin{array}{l} {\bar{r}}_{T}:=\text{E}\left(\text{E}(r_{T}|\vartheta_{T}\right))=\frac{{\bar{\vartheta }}_{T}}{A_{r}}=a\frac{A_{m}}{A_{r}}T+\left(1-a\right)\frac{A_{m}}{A_{r}}E\left(T\right)\;. \end{array}$$

From Equation (11) we also find an expression for the bias corresponding to a stimulus *T*

(12) $$\begin{array}{l} \text{BIAS}r_{T}\;:=\;{\bar{r}}_{T}-T\;=\;-(1-a\frac{A_{m}}{A_{r}})T+\;(1-a)\frac{A_{m}}{A_{r}}\;ET\;. \end{array}$$

Equations (11, 12) directly relate the stimulus *T* to its reproduced value. Expected value and bias of the reproduced stimuli not only depend on the current stimulus *T* but also on the expected value of the stimulus distribution E (*T*). The latter adds an offset to the linear relations. The memory weight *a* contributes to the slope of the relations and thus determines the strength of the regression effect. Values of *a* closer to zero result in stronger regression to mean; for values of *a* closer to one, regression vanishes and reproduction is veridical. As we will see in Section 3, the weight *a* can be constrained by other model parameters to minimize reproduction errors. Regression and range effects are consequences of such optimization efforts.

Expected value and bias of the reproduction according to Equations (11, 12) also depend on the ratio of drifts from both production and reproduction, *A*_*m*_ and *A*_*r*_, respectively. Calculating the expectations

(13) $$\begin{array}{l} E\left({\bar{r}}_{T}\right)=\frac{A_{m}}{A_{r}}E\left(T\right)\;\text{and}\;E\left(\mathrm{BIAS}r_{T}\right)=\left(\frac{A_{m}}{A_{r}}-1\right)E\left(T\right) \end{array}$$

shows that for mismatches between the drifts *A*_*m*_ and *A*_*r*_ we get overall deviations between stimuli *T* and reproductions *r*_*T*_. These non-zero average biases may explain overall over-estimation (for *A*_*m*_∕*A*_*r*_ > 1) and overall under-estimation (*A*_*m*_∕*A*_*r*_ < 1), respectively.

To determine the variance Var (*r*_*T*_) : = Var (*r*∣*T*) in a trial in which the stimulus *T* was presented, we apply the law of total variance and obtain

(14) $$\begin{array}{l} \mathrm{Var}\left(r_{T}\right)=E\left(\mathrm{Var}\left(r_{T}|\vartheta_{T}\right)\right)+\mathrm{Var}\left(E\left(r_{T}|\vartheta_{T}\right)\right)\\ =E\left(\frac{\vartheta_{T}\sigma_{r}^{2}}{A_{r}^{3}}\right)+\mathrm{Var}\left(\frac{\vartheta_{T}}{A_{r}}\right)\\ =\left(\frac{aA_{m}\sigma_{r}^{2}}{A_{r}^{3}}+\frac{a^{2}\sigma_{m}^{2}}{A_{r}^{2}}\right)T\\ +\left(1-a\right)E\left(T\right)\frac{A_{m}\sigma_{r}^{2}}{A_{r}^{3}}\\ +\left[A_{m}^{2}\mathrm{Var}\left(T\right)+\sigma_{m}^{2}E\left(T\right)\right]\frac{a{\left(1-a\right)}^{2}}{A_{r}^{2}\left(2-a\right)}. \end{array}$$

Like the variance of the threshold, also the variance Var (*r*_*T*_) depends on the current stimulus *T* and the statistics of the stimulus distribution given by E (*T*) and Var (*T*). Note that the monotonic relation (14) between stimulus *T* and variance Var (*r*_*T*_) of its reproduction is equivalent to scalar variability.

With formulas (11–14), we have a full characterization of the model linking the stimuli *T* to their reproduced values *r*_*T*_. The description also details the dependence on the different model parameters, i.e., the internal processing. Figure 1 gives examples how formulas (11–14) fit to simulations of the model.

## 3. Results

As displayed in Figure 1, the model described in Section 2 can reproduce the typical psychophysical findings for magnitude estimation: regression effect, range effect, scalar variability, and sequential effects. However, it remains open how we can motivate the choice of parameters that fit the psychophysical findings. The upcoming paragraphs focus on this question.

### 3.1. How to minimize reproduction errors?

Different factors of uncertainty challenge precise magnitude estimation as it is formulated by the model — such as the statistics of the stimuli and internal sources of noise σ_*m*_ and σ_*r*_. How could a subject cope with these noise sources to minimize estimation errors?

For optimal magnitude reproduction one needs to minimize the mean squared error between a stimulus *T* and its reproduced value *r*_*T*_, i.e., $\mathrm{MSE}r=E\left[{\left(r_{T}-T\right)}^{2}\right]$. The mean squared error can be partitioned into a variance and a bias term

(15) $$\begin{array}{l} \mathrm{MSE}r=\mathrm{Var}\left(r\right)+\mathrm{BIAS}r^{2}\;. \end{array}$$

The description of the variance Var (*r*) in Equation (15) depends on the purpose of optimization. In fact, it is not the total variance for the reproduction that should be minimized here. Rather subjects would want to minimize the variability of individual measurements of a particular stimulus E (Var (*r*_*T*_)) = E (Var (*r*∣*T*)); cf. Jazayeri and Shadlen (2010). From Equation (14) the variance E (Var (*r*_*T*_)) is given by

(16) $$\begin{array}{l} \text{E}\left(\text{Var}\left(r_{T}\right)\right)=\left(\frac{A_{m}\sigma_{r}^{2}}{A_{r}^{3}}+\frac{a^{2}\sigma_{m}^{2}}{A_{r}^{2}}\right)\text{E}\left(T\right)\\ \;+\left[A_{m}^{2}\text{Var}\left(T\right)+\sigma_{m}^{2}\text{E}\left(T\right)\right]\frac{a{\left(1-a\right)}^{2}}{A_{r}^{2}(2-a)}\;. \end{array}$$

The term ${\text{BIAS}}_{r}^{2}$ in Equation (15) refers to the mean squared or quadratic mean of all biases in a test range, i.e., $\text{E }{(\text{BIAS}}_{r_{T}}^{2})$. Using Equation (12) it is given by

(17) $$\begin{array}{l} {\text{BIAS}}_{r}^{2}=\text{E}\left({\text{BIAS}}_{r_{T}}^{2}\right)\\ =\text{E}\left\{{\left[-\left(1-a\frac{A_{m}}{A_{r}}\right)T+\left(1-a\right)\frac{A_{m}}{A_{r}}\text{E}\left(T\right)\right]}^{2}\right\}\\ ={\left(1-a\frac{A_{m}}{A_{r}}\right)}^{2}\text{E}{\left(T\right)}^{2}+\left(\frac{A_{m}^{2}}{A_{r}^{2}}(1+a)-2\frac{A_{m}}{A_{r}}\right)\\ \;\left(1-a\right)\text{E}{\left(T\right)}^{2}\;. \end{array}$$

With Equations (16, 17) the MSE_*r*_ reads as follows

(18) $$\begin{array}{l} {\text{MSE}}_{r}=\text{E}\left(\text{Var}\left(r|T\right)\right)+{\text{BIAS}}_{r}^{2}\\ =\left(\frac{A_{m}\sigma_{r}^{2}}{A_{r}^{3}}+\frac{a^{2}\sigma_{m}^{2}}{A_{r}^{2}}\right)\text{E}\left(T\right)+\left[A_{m}^{2}\text{Var}\left(T\right)+\sigma_{m}^{2}\text{E}\left(T\right)\right]\\ \frac{a{\left(1-a\right)}^{2}}{A_{r}^{2}(2-a)}\\ +{\left(1-a\frac{A_{m}}{A_{r}}\right)}^{2}\text{E}{\left(T\right)}^{2}+\left(\frac{A_{m}^{2}}{A_{r}^{2}}(1+a)-2\frac{A_{m}}{A_{r}}\right)\\ \left(1-a\right)\text{E}{\left(T\right)}^{2}. \end{array}$$

Let us explore the possibility that the memory *a* of the system can be adapted to minimize the mean squared error, i.e., $a_{\text{min}}\;:=\min_{a}\left(\mathrm{MSE}r\right)$. Recall that *a* is connected to the slope of the relation between stimulus *T* and its average reproduction ${\bar{r}}_{T}$ and thus determines the strength of the regression effect; cf. Equation (11). To find *a*_min_ we take the first derivative with respect to *a* of Equation (18) and set it to zero

$$\begin{array}{l} 0\;\underline{\underline{!}}\;\frac{\text{d}}{\text{d}a}{\text{MSE}}_{r}\;=\;\frac{\text{d}}{\text{d}a}\text{E }\left(\text{Var}\left(r|T\right)\right)+\frac{\text{d}}{\text{d}a}{\text{BIAS}}_{r}^{2}\\ 0={\left(\frac{A_{m}}{A_{r}}\right)}^{2}\text{Var}\left(T\right)\left[\frac{2}{{\left(2-a\right)}^{2}}-2a\right]+\frac{\sigma_{m}^{2}}{A_{r}^{2}}\\ \text{ E}\left(T\right)\frac{2}{{\left(2-a\right)}^{2}}+2{\left(\frac{A_{m}}{A_{r}}\right)}^{2}\left[a-\frac{A_{r}}{A_{m}}\right]\text{Var}\left(T\right)\\ 0=\left[\text{Var}\left(T\right)+{\left(\frac{\sigma_{m}}{A_{m}}\right)}^{2}\text{E}\left(T\right)\right]\frac{1}{{\left(2-a\right)}^{2}}-\frac{A_{r}}{A_{m}}\text{ Var}\left(T\right). \end{array}$$

Solving *a* = *a*_min_, weobtain

(19) $$a_{\text{min}}=2-\sqrt{\frac{A_{m}}{A_{r}}\left[{\left(\frac{\sigma_{m}}{A_{m}}\right)}^{2}\frac{\text{E}\left(T\right)}{\text{Var}\left(T\right)}+1\right]}\;.$$

**Figure 3:**
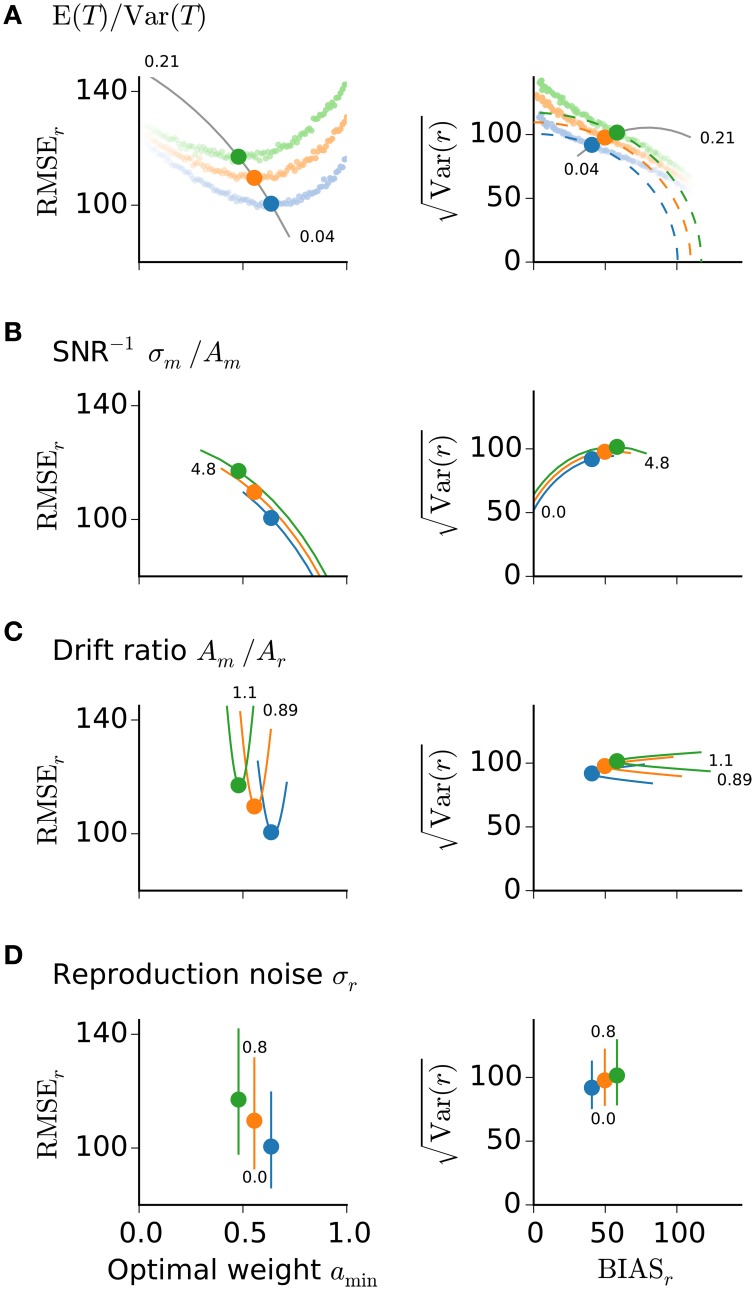
**Reproduction error and model parameters**. Root-mean-squared error (left panels) and its representation on a quarter circle $\sqrt{\mathrm{Var}\left(r\right)}$ vs. BIAS_*r*_ (right panels) are displayed for the optimal memory weight *a*_min_ conditioned on E (*T*)∕Var (*T*) **(A)**, the inverse SNR of measurement σ_*m*_∕*A*_*m*_ **(B)**, the drift ratio *A*_*m*_∕*A*_*r*_ **(C)**, and the noise level σ_*r*_ during reproduction **(D)**. Solid lines show the predictions of *a*_min_ for different values of the respective ratio or parameter. Small numbers mark the range of values. Large dots mark the theoretical predictions from Equation (19) and correspond to the memory weights *a* taken in Figure 1. Colors as in Figure 1. In **(A)** also simulation results are displayed for the three stimulus ranges from Figure 1 and different values of *a* (small dots, fainter colors correspond to smaller values of *a*). The simulations confirm the theoretical predictions for the optimal values *a*_min_.

Simulation results confirm the derivation that led to Equation (19); cf. Figure 3A.

According to Equation (19) a subject may reduce its overall reproduction error by adjusting the strength of regression, depending on the values of three different relations: (i) the drift ratio *A*_*m*_∕*A*_*r*_, which may account for overall biases, cf. Equation (13); (ii) the inverse signal-to-noise ratio (SNR) of the measurement σ_*m*_∕*A*_*m*_, quantifying internal noise; and (iii) the inverse of the index of dispersion (variance-to-mean ratio, Fano factor) of the stimulus distribution E (*T*)∕Var (*T*), characterizing the stimulus distribution, and constituting an external source of uncertainty — in contrast to the other two ratios that are due to internal processing. Note that noise in the reproduction, i.e., σ_*r*_, does not influence *a*_min_, which intuitively makes sense since the update-step of the memory weight *a* precedes the reproduction stage.

### 3.2. Optimality predicts range and regression effects

**Figure 4:**
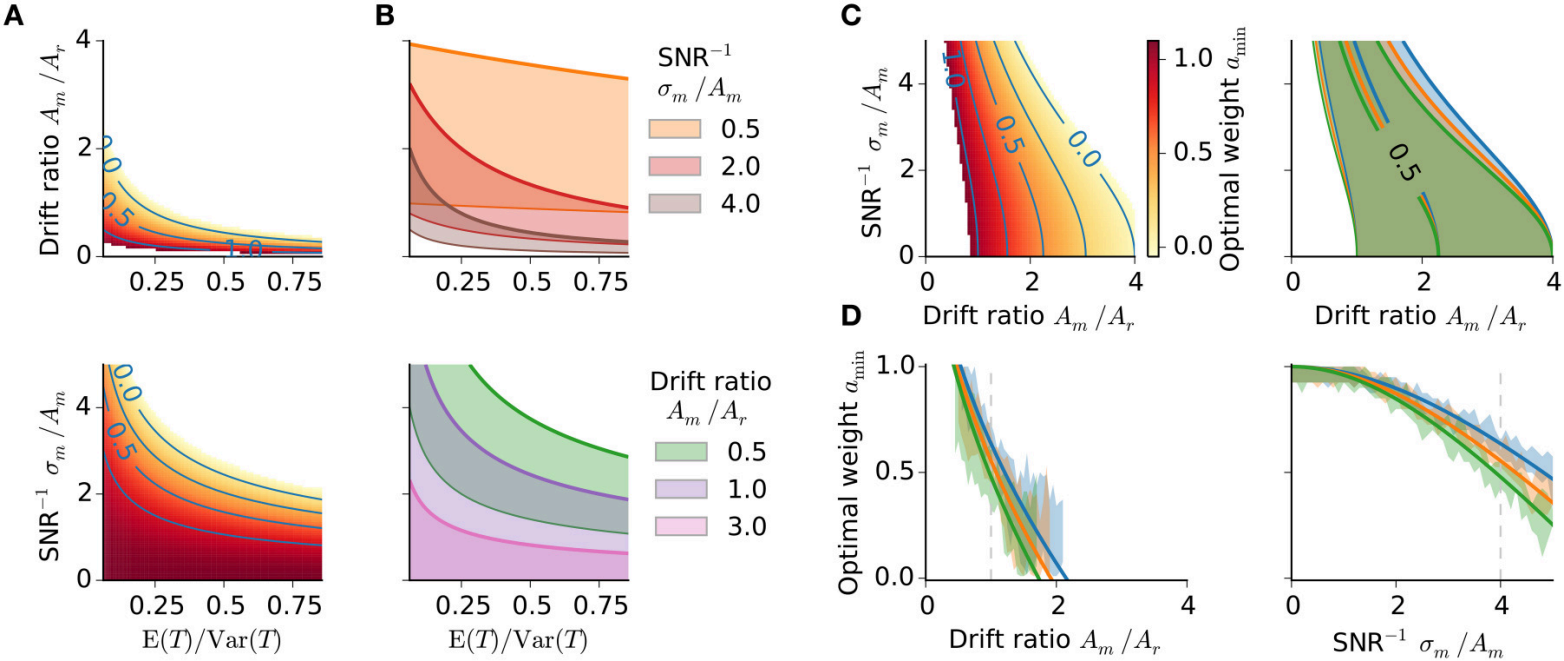
**Optimality characteristics**. **(A,B)** Optimal weight *a*_min_ in dependence on the stimulus distribution. **(A)** Optimal weight *a*_min_ with SNR^−1^ fixed to 4 (*upper panel*) and drift ratio of 1 (*lower panel*), respectively. Same color bar as in (**C**, left). **(B)** Regions of *a*_min_ ∈ (0, 1] for three different SNR^−1^ and drift ratios, respectively; thick colored lines *a*_min_ = 0, thin colored lines *a*_min_ = 1. **(C)** Optimal memory weight *a*_min_ as a function of drift ratio and inverse signal-to-noise ratio. *Left:* Optimal weight *a*_min_ for the stimulus range 494 − 847 ms. *Right:* Regions of *a*_min_ ∈ (0, 1] for each stimulus range from Figure 1. Colors as in Figure 1. **(D)** Optimal weight *a*_min_ as a function of the drift ratio and inverse SNR, respectively, for all three stimulus ranges from Figure 1. Again an inverse SNR of 4 and a drift ratio of 1, respectively, have been used. Gray dashed lines mark those values. Simulation data (1000 runs per stimulus) confirm the theoretical prediction (shaded areas of same color, 10% percentile for minimal MSEs).

To evaluate how the optimal memory weight *a*_min_ depends on the above ratios, let us consider their individual influences on the reproduction error (Figure 3) and determine their interaction (Figure 4). Figure 3 displays the reproduction error as a function of *a*_min_ for different choices of the model parameters. Instead of the mean squared error its square root ${\text{RMSE}}_{r}=\sqrt{{\text{MSE}}_{r}}$ is plotted, which allows for the intuitive visualization of the Pythagorean sum (15) on quarter circles of similar MSE_*r*_-levels (Figure 3, right panels).

The MSE_*r*_ increases with larger ratio E (*T*)∕Var (*T*); Figure 3A. The dependence serves as an explanation of range effects in magnitude estimation, i.e., dependencies on the stimulus statistics, — an experimentally testable prediction (cf. 4). A larger ratio E (*T*)∕Var (*T*) corresponds to a narrower stimulus distribution and thus smaller differences between particular stimuli, which in turn are harder to distinguish. This increases uncertainty about the stimuli, which a subject could balance by increasing regression and hence treat different stimuli as more similar (closer to their mean) as they in fact are. Stronger regression is obtained by letting the memory weight *a*_min_ tend to zero. Note that stronger regression, i.e., smaller *a*_min_, results in a stronger change in the BIAS_*r*_-component compared to the variance component $\sqrt{\text{Var}\left(r\right)}$ (Figure 3A, right panel). Figures 4A,B examines the relation between E (*T*)∕Var (*T*) and the other model parameters with regard to the optimal weight *a*_min_. Only regions with *a*_min_ ∈ (0, 1] are displayed to obtain parameter combinations where optimization is possible. The parameter regions where MSE_*r*_ could be optimized shrink with larger E (*T*)∕Var (*T*) and are further diminished when conditioned on the drift ratio and SNR^−1^ (Figures 4A,B).

Larger measurement noise, i.e., SNR^−^ = σ_*m*_/*A*_*m*_, increases MSE_*r*_ (Figure 3B); to balance this the optimal memory weight *a*_min_ decreases accordingly (Figures 3B, 4C,D). For larger measurement noise, reproduction errors are minimized by increasing regression. The regression effect can thus be interpreted as a strategy to reduce reproduction errors given noisy estimates. In contrast, very precise estimation would lead to veridical judgements about the stimuli. Note, the connection between the inverse SNR and the Weber fraction from psychophysics. Larger SNR^−1^ corresponds to reduced sensory resolution, i.e., lower discriminability, which results in a larger Weber fraction.

The optimal weight *a*_min_ also depends on the drift ratio *A*_*m*_∕*A*_*r*_, which if not equal to one, leads to systematic biases, i.e., overall under- or overestimation; cf. Equation (13), and thus larger MSE_*r*_ (Figure 3C). To compensate for the introduced overall bias (Figure 3C, right panel), drift ratios greater than one require smaller *a*_min_ and drift ratios smaller than one require larger *a*_min_ (Figures 4C,D). Note that the impact of the drift ratio *A*_*m*_∕*A*_*r*_ on *a*_min_ might point in the opposite direction as that of the external and internal uncertainties, E (*T*)∕Var (*T*) and σ_*m*_∕*A*_*m*_, respectively.

In summary, the dependence of *a*_min_ on the noise level during measurement σ_*m*_∕*A*_*m*_ predicts the regression effect and the dependence on the stimulus statistics E (*T*)∕Var (*T*) explains the range effect. The dependence on the ratio of drifts *A*_*m*_∕*A*_*r*_ explains systematic effects like overall over- and underestimation. As already mentioned above noise in the reproduction σ_*r*_ does not affect *a*_min_; cf. Equation (19). Nevertheless, MSE_*r*_ gets increased with larger reproduction noise (Figure 3D). Noise in measurement and reproduction therefore differently affects the bias and the variance of stimulus reproduction.

### 3.3. Explaining sequential effects

A fourth class of psychophysical characteristics that was mentioned in the introduction was not considered so far, i.e., effects related to stimulus order (Cross, 1973; Petzschner et al., 2015). Due to the trial-by-trial update rule incorporated in the model, previous trials unavoidably affect the reproduction of the current stimulus. Figure 1C exemplifies this via the biases for a particular stimulus conditioned on the stimulus in the previous trial. In general the bias for the current stimulus is proportional to the immediately preceding stimulus. To evaluate this effect analytically, let us reconsider Equation (7). We take out trial *n* − 1 from the sum and proceed in similar steps to the derivation in Section “2.2,” which lead to Equation (9) and finally to Equation (11). The average response to stimulus *T* given that stimulus *T*_*n*−1_ was presented in the preceding trial is obtained from

(20) $$\begin{array}{l} {\bar{r}}_{T,T_{n-1}}=a\frac{A_{m}}{A_{r}}T+a(1-a)\frac{A_{m}}{A_{r}}\Delta T_{n-1}+(1-a)\frac{A_{m}}{A_{r}}\text{E}\left(T\right)\\ ={\bar{r}}_{T}+a(1-a)\frac{A_{m}}{A_{r}}\Delta T_{n-1}\;. \end{array}$$

We express the previous stimulus relative to the mean E (*T*) here, i.e., Δ*T*_*n*−1_ = *T*_*n*−1_ − E (*T*). The effect of the previous onto the current trial, we evaluate by the corresponding BIAS

(21) $${\text{BIAS}}_{T_{n},T_{n-1}}={\bar{r}}_{T_{n},T_{n-1}}-T\;=\;{\text{ BIAS}}_{r_{T}}+a(1-a)\frac{A_{m}}{A_{r}}\Delta T_{n-1}\;.$$

Thus, when a stimulus value *T*_*n*−1_ larger than E (*T*) was presented in the previous trial a positive term is added to BIAS_*rT*_. For a stimulus *T*_*n*−1_ < E (*T*) the term is negative and the bias will become smaller (Figure 1C).

## 4. Discussion

The model introduced in the present paper describes magnitude estimation as a two-stage process, measurement and reproduction, consisting of noisy integrators linked by an internal reference (*implicit standard* or *prior*) that is updated on a trial-by-trial basis.

Trial-by-trial update rules have been used by others to explain aspects of magnitude estimation (Hellström, 2003; Dyjas et al., 2012; Bausenhart et al., 2014) and are also at the core of the Bayesian model by Petzschner and Glasauer (2011), where such updating is used to adjust prior knowledge about the stimulus distribution. Iterative updating in the present model estimates the moments of the stimulus distribution to form an internal reference. At least humans are known to be able to maintain (Morgan et al., 2000) and to quickly adapt such an internal reference (Berniker et al., 2010).

Noisy integrative processes well describe decision-making at the behavioral level (Brunton et al., 2013). Moreover, several brain regions show noisy integration during decision-making (Shadlen and Newsome, 2001; Liu and Pleskac, 2011; Shadlen and Kiani, 2013; Hanks et al., 2015) at least at the population level. Whether noisy integration is generated by ramp-like noisy integration in single neurons has been questioned recently (Latimer et al., 2015). In any case, the present model suggests that noisy integration is also crucial to non-binary cognitive demands such as the representation and processing of magnitudes.

### 4.1. Connection to psychophysical effects of magnitude estimation

The presented model reproduces the main behavioral characteristics of magnitude estimation Figure 1; (Petzschner et al., 2015): Estimates tend toward the mean (regression effect) and this effect scales with the range of stimuli chosen (range effect). Errors monotonically increase with the size of the stimulus (scalar variability). In addition, the sequence in which stimuli are presented influences magnitude judgments. Such sequential effects are by design captured by the model due to the trial-by-trial update of the internal reference. The major insight from this paper therefore is that iterative updating can explain regression and range effects (see also Bausenhart et al., 2014). As such both effects are consequences of strategies to minimize reproduction errors. With larger uncertainty about the stimuli, stronger regression helps to minimize reproduction errors and hence optimizes judgements. Uncertainty may stem from both internal and external sources, whose influence can be evaluated separately by the presented approach.

Internal noise is quantified by the signal-to-noise ratio (SNR) during measurement, i.e., inverse SNR in Equation (19), which corresponds to the Weber fraction in psychophysics and thus the discrimination abilities of a subject. Weber fractions depend on the stimulus modality and are subject-specific. “Modality effects” and individual differences are well known in interval timing literature (Shi et al., 2013). Cicchini et al. (2012) showed that percussionists precisely reproduce temporal intervals and display very weak regression effects in contrast to normal subjects. In addition, the results depended on stimulus modality. For all subject groups, performance was better when intervals were given by auditory rather then visual stimulation. The results of Cicchini et al. (2012) are in line with the present model due to the connection between regression effect and Weber fraction. To explain their experimental data, Cicchini et al. (2012) proposed a Bayesian model that included information about the discrimination abilities (Weber fractions) and obtained very similar results to the present work. Increasing SNR (decreasing Weber fraction) during measurement would require adjusting the drift rate *A*_*m*_ such that it is as large as possible compared to the noise σ_*m*_. However, the drift rate *A*_*m*_ will be limited from above by neuronal and network processes, and related time constants (Murray et al., 2014). Analogous constraints were derived by Cicchini et al. (2012) on the width of the prior distribution. Parkinson patients tested off of their medication display strong regression effects (Malapani et al., 1998, 2002). In addition, the precision of the responses is reduced. This is in line with the present model, since stronger regression is predicted with reduced precision (i.e., increased variance or reduced SNR).

External uncertainty is due to stimulus context, i.e., the statistics of the stimuli, which is quantified by the ratio between mean and variance of the stimulus distribution in the present model. Larger ratios (narrower stimulus distributions) should lead to stronger regression. Intuitively this means that the width of the stimulus distribution becomes small compared to its mean and individual stimuli can not be discriminated anymore, hence uncertainty increases. The regression effect counteracts this by treating different stimuli similar to their mean. Note the similarity to the Weber-Fechner law, which predicts decreased discriminability with larger stimuli. In line with this view, more difficult magnitude estimation tasks should display stronger regression effects (Teghtsoonian and Teghtsoonian, 1978; Petzschner et al., 2015).

Systematic over- or underestimation are often found in magnitude estimation experiments (for examples see Jazayeri and Shadlen, 2010; Petzschner and Glasauer, 2011; Cicchini et al., 2012). Such differences may, e.g., occur due to attentional and subject-related factors. In the model this would be attributed to differences in the drift rates from measurement and reproduction. Note that only differences are important, absolute scales of (neural) processing (Kiebel et al., 2008; Murray et al., 2014) are not crucial as long as they are similar across processing stages.

The standard deviation is a monotonically increasing function of the stimulus strength in the model presented here; cf. Equation (14) and Figure 1B. As such the model is in line with the Weber-Fechner law (scalar variability). However, the Weber-Fechner law predicts a linear increase of variability (standard deviation) as a function of magnitude. According to Equation (14) the increase of the standard deviation is sub-linear (square root) in the present model. This sub-linearity may be rather weak (cf. simulation data and theoretical predictions in Figure 1B) and thus may still be in line with experimental data, i.e., differentiating between linearity and weak sub-linearity may be hard from real data. Certain extensions of the model may help to obtain a linear relation. One possibility is introducing a drift ratio *A*_*m*_∕*A*_*r*_ that scales with the stimulus *T*. Whether scalar variability applies to magnitude estimation without restrictions and across all ranges is not clear. This question is, for example, still a matter of debate in interval timing literature, where non-scalar variability has been reported for specific tasks or situations (like timing while counting or singing; Hinton and Rao, 2004; Grondin and Killeen, 2009).

### 4.2. Predictions

The formulation of the optimal memory weight *a*_min_ according to Equation (19) allows for a number of experimentally testable predictions: (i) Reproduced magnitudes should depend on the stimulus distribution. The experimental studies by Jazayeri and Shadlen (2010), Petzschner and Glasauer (2011), Cicchini et al. (2012) only increased the mean of the stimulus distribution between ranges, which would increase the mean-to-variance ratio and predict stronger regression, i.e., a decrease in *a* (cf. Figure 1A). Stimulus distributions with the same mean but larger variances should result in less regression. Indeed, for their experiments on range effects in loudness and distance estimation, Teghtsoonian and Teghtsoonian (1978) varied the width of the stimulus distribution instead of the mean. They found increasing power exponents with wider stimulus distributions. (ii) Regression to the mean should depend on the discrimination abilities of the individual. Subjects with precise perception of the stimulus magnitude under investigation should show less regression than subjects with reduced abilities; (e.g., Cicchini et al., 2012). This should depend on stimulus modality (Cicchini et al., 2012; Shi et al., 2013) and change with training for a specific task. (iii) Seldom stimuli with a low probability of occurrence and with a magnitude way below or way above the stimulus distribution, should not influence the internal reference. (iv) For strong regression the convergence dynamics of the reference should be much slower then for subjects showing weak regression. The influence of previous stimuli should correlate with the level of regression as well as updating of the references after changing the stimulus distribution within an experimental session.

### 4.3. Connection to bayesian models of magnitude estimation

Magnitude estimation has been successfully explained by Bayesian models (Jazayeri and Shadlen, 2010; Petzschner and Glasauer, 2011; Cicchini et al., 2012; Petzschner et al., 2015). The relation between the present work and the Bayesian approaches is not investigated in detail. Nevertheless, some connections shall be discussed. An equivalence between drift-diffusion models and Bayesian frameworks has been described for modeling perceptual decision making (Bitzer et al., 2014) and may also be possible to be established for the model presented here. The measurement phase results in an internal estimate *m* of a stimulus *T* drawn from a likelihood distribution *p*(*m* ∣ *T*). The reproduction process gives a posterior estimate, the reproduced stimulus *r*, drawn from the distribution *p*(*r* ∣ *m*). It has to be explored, however, (i) whether the update rule Equation (6) implements a way of connecting both the likelihood *p*(*m* ∣ *T*) and the posterior *p*(*r* ∣ *m*) in a Bayes-optimal way; (ii) in how far the update-rules used here and in Petzschner and Glasauer (2011) correspond to each other; and (iii) if the remarkable agreement between the present results and that of the Bayesian description by Cicchini et al. (2012) indicates more than conceptual conformity, i.e., the connection between minimization of reproduction errors and strength of regression, and their modulation by the precision of sensory representations and by the stimulus distribution.

In general, interpreting the regression effect as a means of error minimization shares similarities with concepts like the free-energy principle (Friston, 2010) and information maximization (Linsker, 1990). Error minimization corresponds to the idea of minimizing surprise (free energy) or prediction error and hence maximizing reward.

### 4.4. Neural implementation?

Noisy integrative activation patterns are found in several brain regions during decision-making tasks (for a recent review see, e.g., Shadlen and Kiani, 2013). It remains open, however, if such patterns are also present during magnitude estimation as proposed by the model presented here. Neurons sensitive to elapsed time have been shown, for instance, in parietal cortex (Leon and Shadlen, 2003), hippocampus (MacDonald et al., 2011; Sakon et al., 2014), and basal ganglia (Jin et al., 2009; Mello et al., 2015). Neurons in rat hippocampus code for distance covered (Kraus et al., 2013). Single neurons in rat prefrontal cortex show temporally modulated activation patterns during interval timing (Kim et al., 2013; Xu et al., 2014). Such single cell activation patterns may form a set of basis functions to drive noisy integrative processes (c.f. Ludvig et al., 2008; Goldman, 2009; Mello et al., 2015) and may arise in neural networks with balanced excitation and inhibition (Simen et al., 2011), from firing rate adaptation (Reutimann et al., 2004), or from single neuron dynamics (Durstewitz, 2003) — although it has been questioned recently if ramp-like activity is present in single cells (Latimer et al., 2015). It is, furthermore, conceivable to obtain processes akin to noisy integration from state dependent networks (Karmarkar and Buonomano, 2007; Buonomano and Laje, 2010; Laje and Buonomano, 2013). Another question that arises when thinking about a neural implementation of the model introduced in this paper concerns the implementation of the adaptive threshold. It has been suggested from network models of perceptual decision making that adaptive thresholds for noisy integrative processes may be implemented with the help of synaptic plasticity in cortico-striatal circuits (Lo and Wang, 2006; Wei et al., 2015).

## 5. Conclusions

The model presented in this paper describes magnitude estimation as two-stages of noisy integration linked by an iteratively updated internal reference memory. Behavioral characteristics well known from magnitude estimation experiments are not only reproduced but also explained as a means of minimizing errors given estimates corrupted by internal and external sources of noise. This paper thus shows that noisy integrative processes may be crucial for cognitive demands beyond perceptual decision making, such as the processing of magnitudes — suggesting an overall computational principle and likely common neural mechanisms that we use to perceive and interpret our environment.

## Author contributions

The author confirms being the sole contributor of this work and approved it for publication.

### Conflict of interest statement

The author declares that the research was conducted in the absence of any commercial or financial relationships that could be construed as a potential conflict of interest.
